# Molecular and Biomechanical Clues From Cardiac Tissue Decellularized Extracellular Matrix Drive Stromal Cell Plasticity

**DOI:** 10.3389/fbioe.2020.00520

**Published:** 2020-05-29

**Authors:** Gabriel Romero Liguori, Tácia Tavares Aquinas Liguori, Sérgio Rodrigues de Moraes, Viktor Sinkunas, Vincenzo Terlizzi, Joris A. van Dongen, Prashant K. Sharma, Luiz Felipe Pinho Moreira, Martin Conrad Harmsen

**Affiliations:** ^1^Department of Pathology and Medical Biology, University Medical Center Groningen, University of Groningen, Groningen, Netherlands; ^2^Instituto do Coração (InCor), Hospital das Clinicas HCFMUSP, Faculdade de Medicina, Universidade de São Paulo, São Paulo, Brazil; ^3^Department of Biomedical Engineering, University Medical Center Groningen, University of Groningen, Groningen, Netherlands

**Keywords:** hydrogels, extracellular matrix proteins, tissue engineering, regenerative medicine, biocompatible materials, biomimetic materials, tissue scaffolds, tissue decellularization

## Abstract

Decellularized-organ-derived extracellular matrix (dECM) has been used for many years in tissue engineering and regenerative medicine. The manufacturing of hydrogels from dECM allows to make use of the pro-regenerative properties of the ECM and, simultaneously, to shape the material in any necessary way. The objective of the present project was to investigate differences between cardiovascular tissues (left ventricle, mitral valve, and aorta) with respect to generating dECM hydrogels and their interaction with cells in 2D and 3D. The left ventricle, mitral valve, and aorta of porcine hearts were decellularized using a series of detergent treatments (SDS, Triton-X 100 and deoxycholate). Mass spectrometry-based proteomics yielded the ECM proteins composition of the dECM. The dECM was digested with pepsin and resuspended in PBS (pH 7.4). Upon warming to 37°C, the suspension turns into a gel. Hydrogel stiffness was determined for samples with a dECM concentration of 20 mg/mL. Adipose tissue-derived stromal cells (ASC) and a combination of ASC with human pulmonary microvascular endothelial cells (HPMVEC) were cultured, respectively, on and in hydrogels to analyze cellular plasticity in 2D and vascular network formation in 3D. Differentiation of ASC was induced with 10 ng/mL of TGF-β1 and SM22α used as differentiation marker. 3D vascular network formation was evaluated with confocal microscopy after immunofluorescent staining of PECAM-1. In dECM, the most abundant protein was collagen VI for the left ventricle and mitral valve and elastin for the aorta. The stiffness of the hydrogel derived from the aorta (6,998 ± 895 Pa) was significantly higher than those derived from the left ventricle (3,384 ± 698 Pa) and the mitral valve (3,233 ± 323 Pa) (One-way ANOVA, *p* = 0.0008). Aorta-derived dECM hydrogel drove non-induced (without TGF-β1) differentiation, while hydrogels derived from the left ventricle and mitral valve inhibited TGF-β1-induced differentiation. All hydrogels supported vascular network formation within 7 days of culture, but ventricular dECM hydrogel demonstrated more robust vascular networks, with thicker and longer vascular structures. All the three main cardiovascular tissues, myocardium, valves, and large arteries, could be used to fabricate hydrogels from dECM, and these showed an origin-dependent influence on ASC differentiation and vascular network formation.

## Introduction

Decellularized organ-derived extracellular matrix (dECM) has been used for many years in tissue engineering and regenerative medicine (Conklin et al., 2002; Dahl et al., 2003; Schenke-Layland et al., 2003). Decellularization of the extracellular matrix can be achieved through different physical, chemical or biological processes, including freeze/thaw cycles, use of organic detergents, and mild treatment with proteolytic enzymes (Crapo et al., 2011; Hrebikova et al., 2015; Keane et al., 2015; Leonel et al., 2017; Kawecki et al., 2018). Commonly, different processes are combined or used sequentially, so as to decrease the damage caused by each of them to the extracellular matrix but, at the same time, to potentiate the decellularization protocol (Crapo et al., 2011; Kawecki et al., 2018). In dECM, both the structure and biochemical composition of the original tissue are largely retained which renders dECM a promising candidate for cell culture and tissue engineering (Hoshiba et al., 2010, 2016; Cigliano et al., 2012; Petersen et al., 2012; Poornejad et al., 2016). Also, regeneration-related processes such as recruitment of progenitor cells, induction of cell migration and proliferation, and M2 polarization of in macrophages appear to occur upon *in vivo* administration of dECM (Reing et al., 2009; Agrawal et al., 2011; Brown and Badylak, 2014; Dziki et al., 2017). In general, the large(r) macromolecules of ECM such as polysaccharides (glycosaminoglycans and proteoglycans) as well as constructive proteins (collagens, basement membrane proteins, and fibronectin to mention a few) remain after decellularization because of their size and their intermolecular crosslinks. Smaller ECM constituents such as growth factors, chemokines, and other small signaling molecules are largely washed out.

Originally, decellularization of whole organs was intended to reseed stem cells or parenchymal cells to recreate the organ. More recently, clinical interest shifted to use dECM, as powder or as hydrogel, for repair and regeneration purposes of organ damage more than as replacement therapy (Adam Young et al., 2011; Wolf et al., 2012; Mercuri et al., 2013; Fu et al., 2016; Ungerleider et al., 2016; Saldin et al., 2017). Hydrogels derived from dECM are tuneable with respect to biochemical parameters via loading with growth factors, stem cells while their physical parameters such as stiffness and viscoelasticity are tuneable too (Adam Young et al., 2011; DeQuach et al., 2012; Seif-Naraghi et al., 2012; Ungerleider et al., 2015; Wu et al., 2015). The use of 3D bioprinting, though in its infancy with dECM-derived hydrogels, enables to print predetermined shapes and geometries of factor and cell-loaded gels.

Traditionally, cardiovascular tissue engineering has focused on replacement tissue for coronary arteries, cardiac valves as well as left ventricular myocardium with no definitive success for any of these three (Singelyn et al., 2009, 2012; Seif-Naraghi et al., 2010, 2012; Duan et al., 2011; Johnson et al., 2011, 2014; O'Neill et al., 2013; Grover et al., 2014; Pok et al., 2014; Russo et al., 2015; Ungerleider et al., 2015, 2016; Kappler et al., 2016; Stoppel et al., 2016; Wassenaar et al., 2016a,b; Efraim et al., 2017; Fercana et al., 2017; Jang et al., 2017; Wang et al., 2017; Seo et al., 2018). Most of these efforts used collagen or single ECM molecule-based scaffolds besides a host of synthetic polymer materials. We reasoned that regeneration of damaged specific cardiac compartments (myocardium, valve, or arteries) would benefit from the use of dECM hydrogels derived from that same compartment. In other words, we hypothesized that dECM-derived hydrogels from myocardium, valves, and aorta would differ in biological and physical features.

## Methods

**Figure 1:**
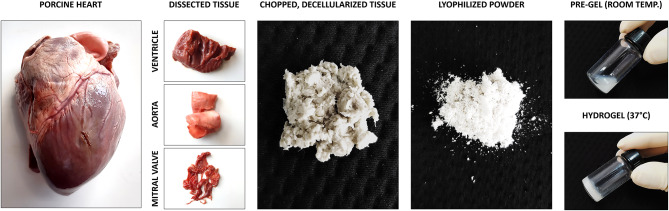
Illustrative overview of the methods used to produce hydrogels derived from decellularized extracellular matrix.

An illustrative overview of the methods used for the fabrication of dECM-derived hydrogels is presented in Figure 1. Detailed description is described below.

### Extracellular Matrix Decellularization and Characterization

#### Decellularization Protocol

Porcine hearts (12-week old pigs) were bought from a local slaughterhouse (Kroon Vlees, Groningen, Netherlands) and dissected to separate the left myocardial tissue, the mitral valve, and the aorta. The tissues were washed at room temperature (RT) in phosphate buffer saline (PBS) and triturated in a commercial blender until pieces were smaller than 1 mm. Following a second wash in PBS, tissue material was sonicated for 1 min at 100% power, washed a third time and incubated in 0.05% trypsin (Gibco, Thermo Fisher Scientific, Waltham, USA) in PBS at 37°C for 3 h under constant shaking. After trypsin treatment, tissue material was washed again with PBS and frozen at −20°C overnight. After thawing, tissue material was incubated in demineralized water (dH_2_O) for 3 h and then in saturated sodium chloride solution (NaCl, 6M) for another 3 h, both steps at 37°C while shaking continuously. This followed by a wash with 70% ethanol for 10 min and dH_2_O for another 10 min. Afterwards, tissue material treated with 1% sodium dodecyl sulfate (SDS) (Sigma-Aldrich, St. Louis, USA) in water for 12 h, washed three times with dH_2_O, incubated with 1% Triton X-100 (Sigma-Aldrich, St. Louis, USA) in water for 12 h, washed three times with dH_2_O, incubated with 1% sodium deoxycholate (Sigma-Aldrich, St. Louis, USA) in water for 12 h and, again, washed three times with dH_2_O, all steps while shaking continuously. After these detergent treatments, tissue material was incubated for 24 h with DNAse solution [30 μg/ml DNAse (Worthington Biochemical Corporation, Lakewood, USA), 1.3 mM MgSO_4_, 2 mM CaCl_2_] at 37°C and continuous shaking. After DNAse treatment, the decellularized tissue remainder i.e., extracellular matrix (dECM) was washed three times with dH_2_O and stored at 4°C in 1% penicillin/streptomycin (#15140122, Gibco Invitrogen, Carlsbad, USA) in sterile PBS.

The dECM characterization was comprised of quantification of residual nuclei, genomic DNA, histochemical stainings (Hematoxylin/Eosin and Movat's Pentachrome), quantification of GAG contents and identification of proteins by mass spectrometry.

#### Residual Nuclei and DNA Quantification

For evaluating residual nuclei, native and dECM samples were stained with 4′,6-diamidino-2-phenylindole (DAPI) (4 μg/ml; #D9542-5MG, blue; Sigma-Aldrich, St. Louis, USA) and visualized with an Evos FL fluorescence microscope system (Thermo Fisher Scientific, Waltham, United States).

To isolate genomic DNA freeze-dried native and dECM samples (*n* = 3) were weighed and separated as samples of 10–15 mg in a 1.5 mL microfuge tube. Each sample was digested at 55°C in a solution containing 5 μL proteinase K (#3115828001, Roche, Basel, Switzerland), 50 μL 10% SDS, and 500 μL SE-buffer (75 mM NaCl; 25 mM EDTA; pH 8.0) overnight. Following the enzymatic digestion, 222 μL of 6 M NaCl and 777 μL chloroform were added and samples thoroughly shaken on a top-over-top rotator at room temperature (RT) for 1 h and centrifuged at 10,000 × g at 20°C for 10 min. After centrifugation, the upper layer was transferred to a new tube, and an equal volume of ice-cold isopropanol was added and gently mixed until DNA precipitated (if any). The DNA was collected by centrifugation at 10,000 × g at 4°C for 15 min. The DNA pellet was washed with 0.5 mL ice-cold 70% ethanol centrifuged at 10,000 × g at 4°C for 5 min. Upon removal of the supernatant, the pellet was left to air-dry in RT. The DNA was dissolved in 100 μL 10 mM Tris, 0.1 mM EDTA, pH 8.0 at 55°C and quantified with NanoDrop equipment (Thermo Scientific, Hemel Hempstead, United Kingdom).

#### Histochemical Analysis

For histochemical stainings, samples of native and dECM were fixed with 4% paraformaldehyde (PFA) in PBS for 1 h, washed with PBS twice, and embedded in paraffin. Five micrometer sections were mounted on glass microscope slides, deparaffinized, stained with Hematoxylin/Eosin (HE) or Movat's pentachrome stain, mounted with mounting medium and visualized with a light microscope (DM IL, Leica Microsystems, Wetzlar, Germany). All samples were stained simultaneously to rule out the influence of staining variations among groups.

#### GAG Quantification

The concentration of sulfated glycosaminoglycans (sGAG) was measured using the 1,9-dimethylmethylene blue (DMMB) assay, according to the protocol of Farndale et al. (1982). Briefly, dECM samples (*n* = 3) were weighed and separated as samples of 10–15 mg in a 1.5 mL microfuge tube. Each sample was digested at 55°C in a solution containing 5 μL proteinase K (#3115828001, Roche, Basel, Switzerland), 50 μL 10% SDS, and 500 μL SE-buffer (75 mM NaCl; 25 mM EDTA; pH 8.0) overnight. After digestion, DMMB staining solution (10:1 to the initial volume) was added to the samples and excitation was measured at 525 nm and 595 nm using a spectrophotometer. A titration curve of serially diluted chondroitin sulfate C (#C4384-250 mg, Sigma-Aldrich, St. Louis, USA) was used as control.

#### Mass Spectrometry

Samples (*n* = 3) of dECM from the three different types of cardiovascular tissue, resp. the left ventricle, mitral valve, and aorta, were submitted to mass spectrometry analyses to determine the protein composition in the decellularized tissue. Lyophilized dECM was milled to a fine powder and digested in pepsin (#P6887; >3,200 IU; Sigma-Aldrich, St. Louis, USA) solution. The digested solution was ultrafiltrated using centrifugal filter units (Millipore, Billerica, USA) to remove undigested tissue debris. Liquid chromatography-tandem mass spectrometry (LC-MS/MS) analysis was performed using an integrated system composed of nano-LC (Tempo nano-LC system, MDS SCIEX, Ontario, Canada) and a Quadrupole-TOF MS/MS spectrometer (QStar Elite, Applied Biosystems, Foster City, USA) equipped with a nano-electrospray ionization source at an ion spray voltage of 2.3 keV. Solutions containing pepsin-digested protein fragments were injected into the nano-LC-MS/MS system and then separated on a Zorbax 300SB-C18 capillary column (Agilent Technologies, Palo Alto, USA). The loaded samples were eluted with a 2–35% gradient of solvent B for 30 min, then a 35–90% gradient for 10 min, followed by 90% solvent B for 5 min, and finally 5% solvent B for 15 min at a flow rate of 300 nL/min. Solvent A consisted of water/acetonitrile (98/2 [v/v]) and 0.1% formic acid, and solvent B consisted of water/acetonitrile (2/98 [v/v]) and 0.1% formic acid. Data acquisition and processing were performed with Analyst QS 2.0 software (Applied Biosystems, Foster City, United States). Generated MS/MS data were compared to the UniProtKB database for *Sus scrofa*.

### dECM Hydrogels Fabrication and Characterization

#### dECM Gelation

For dECM gelation, the decellularized extracellular matrices of the three cardiovascular tissues were lyophilized and milled to a fine powder. The resulting dECM powder was digested with pepsin (#P6887; >3,200 IU; Sigma-Aldrich, St. Louis, USA) in hydrochloric acid solution (20 mg/mL dECM, 2 mg/mL pepsin, 0.01 M HCl) for 6 h at RT and constant stirring. After digestion, the solution pH was raised with sodium hydroxide (1/10 of solution volume, 0.1 M NaOH) and the electrolytes equilibrated with PBS (1/10 of solution volume, 10x PBS). The resulting solution was stored in liquid form (pre-gel) at 4°C or transformed into hydrogel by warming to 37°C for 1 h.

#### Scanning Electron Microscopy of dECM Hydrogels

Hydrogels derived from all the three different tissues were gelated and freeze-dried in a lyophilizer for 3 days. Samples were, then, analyzed with a tabletop scanning electron microscope (SEM, Hitachi TM3000, Hitachi High-Technologies, Japan). Images were acquired using 5 kV and 100X augmentation. The same settings were used for all the three specimens.

#### Biomechanical Properties of dECM Hydrogels

Hydrogels derived from the left ventricle, mitral valve, and aorta were subjected to compressive loading using a low load compression tester (LLCT), as described previously with minor modifications (Sharma et al., 2011; Peterson et al., 2013; Nibourg et al., 2015). Two hundred microliter of the hydrogel was placed inside polydimethylsiloxane (PDMS) donut-shaped support on top of a microscope glass slide, which were both placed on the load cell. First, gel thickness was measured by letting the stainless-steel plunger (diameter, 25 mm) drop down till it touched the glass surface; this position was recorded as the bottom of the gel. Then the gel was placed under the plunger and the top of the gel was determined, the difference in position between the top and bottom of the gel was taken as the thickness. Plunger dropped at a speed of 5 μm/s until it experienced a counterforce of 0.0981N and this was defined as touch. Next, the gel was deformed by 20% (strain = 0.2) i.e., to 80% of its original thickness within 1 s (strain rate of 0.2 s^−1^) and the plunger was kept in this position for another 100 s. Gel stiffness was determined during the first second of experiment i.e., while the plunger was deforming the gel as the slope of the stress-strain curve. During the next 100 s the strain was kept constant (at 0.2) while the stress was observed to continuously decrease with time [σ*(t)*]. This stress relaxation at constant strain is proof that the gels are not elastic but viscoelasticity in nature. The viscoelastic properties were determined by dividing the decreasing stress with constant strain (0.2) to get *E(t) (*=*((t)/0.2)*. A generalized Maxwell model was fit to *E(t)* in the form of Equation (1).

(1) $$\begin{array}{r} E\left(t\right)=E_{1}e^{\frac{-t}{\tau_{1}}}+E_{2}e^{\frac{-t}{\tau_{2}}}+E_{3}e^{\frac{-t}{\tau_{3}}}\ldots \ldots \ldots E_{i}e^{\frac{-t}{\tau_{i}}} \end{array}$$

Each Maxwell element corresponds to one $E_{i}e^{\frac{-t}{\tau_{i}}}$ term in Equation (1) where *i* varies from 1 to n. A Maxwell element is characterized by its modulus i.e., *E*_*i*_ and relaxation time constant i.e., τ_*i*_ for which it remains active. Model fitting was performed with the Microsoft Excel 2016 Solver module, imposing positive values for physical relevance. Fitting started with one Maxwell element with an addition of a new element until the Chi-square (error function) kept on decreasing. Each element was assigned with a relative importance (*RI*_*i*_) within each experiment on the basis of the value of its spring constant, *E*_*i*_, and calculated using (Equation 2).

(2) $$\begin{array}{r} RI_{i}=100.\frac{E_{i}}{\left(E_{1}+E_{2}+\ldots E_{i}\right)} \end{array}$$

In order to average replicate measurements and compare different hydrogels based on their viscoelasticity, each Maxwell element was assigned to a relaxation time range. Four log scale-based relaxation time ranges were defined i.e., 0–1 s, 1–10 s, 10–100 s, and 100–1,000 s.

### *In vitro* Influence of dECM Hydrogels on Mesenchymal and Endothelial Cells in 2D and 3D Cultures

#### Adipose Tissue-Derived Stromal Cell Culture and Differentiation on the dECM Hydrogels

Human ASC were isolated as described previously (Tuin et al., 2010). Briefly, human abdominal fat was obtained by liposuction, washed with phosphate-buffered saline (PBS) and digested enzymatically with 0.1% collagenase A (Roche Diagnostic, Mannheim, Germany) in PBS with 1% bovine serum albumin (BSA; Sigma-Aldrich, Boston, USA). The tissue was shaken constantly at 37°C for 2 h. After this, the digested tissue was mixed with 1% PBS/BSA, filtered, centrifuged and the cell pellet was resuspended in Dulbecco's Modified Eagle's Medium (DMEM; #12-604F, Lonza, Basel, Switzerland) with 10% fetal bovine serum (FBS; #F0804, Sigma-Aldrich, Missouri, United States), 1% penicillin/streptomycin (#15140122, Gibco Invitrogen, Carlsbad, USA), and 1% L-glutamine (#17-605E, Lonza Biowhittaker, Verviers, Belgium). Cells were cultured at 37°C in a humidified incubator with 5% CO_2_. The medium was refreshed every 2 days. Confluent cells were passed at a ratio of 1:3.

To assess the influence of hydrogel surfaces on the proliferation and differentiation of ASCs, wells of 24-well plates were covered with 200 μL of the pre-gel solutions from the three different ECM sources and allowed to gelate at 37°C for 1 h. ASC (P3 to 5) were seeded at 10,000 cells/cm^2^ on the dECM hydrogels. Cells were cultured in Dulbecco's modified Eagle's medium (DMEM, Lonza, Verviers, Belgium) supplemented with 10% fetal bovine serum (FBS) (Thermo Scientific, Hemel Hempstead, United Kingdom), 1% L-glutamine (Lonza, Verviers, Belgium), and 1% penicillin/streptomycin (Gibco Invitrogen, Carlsbad, USA) at 37°C in a humidified incubator with 5% CO_2_. As a control, ASC were seeded on tissue culture plastic in the same medium and at the same density. Seeded ASCs were stimulated with 10 μg/ml TGFβ-1 (PeproTech, London, United Kingdom) while non-stimulated cultures served as control. Stimulation was for 7 days and the medium was refreshed every 2 days.

Cells were washed with PBS and fixed with 2% paraformaldehyde in PBS for 30 min, washed with PBS twice, permeabilized with 0.5% Triton X-100 in PBS for 15 min and blocked with 1% BSA and 5% donkey serum solution in PBS for 15 min to avoid non-specific binding. Myogenic differentiation was assessed by staining with rabbit anti-SM22α primary antibody (1:800; Abcam, Cambridge, United Kingdom) for 2 h, washing with 0.05% Tween in PBS and detection with donkey anti-rabbit Alexa Fluor® 647 secondary antibody (1:800; red; Abcam, Cambridge, United Kingdom) for 1 h at room temperature. The cytoskeleton was stained by Phalloidin Alexa Fluor® 488 (1:400; green; ThermoFisher, Waltham, USA) and nuclei were stained with DAPI (4 μg/ml; #D9542-5MG, blue; Sigma-Aldrich, St. Louis, USA). SM22α expression was calculated by the normalized total cell fluorescence (CTCF) method as previously described (McCloy et al., 2014; Liguori et al., 2019), and plotted as the fold-change relative to the non-stimulated tissue culture plastic control. Briefly, the corrected total cell fluorescence (CTCF) is calculated as the integrated density (ID) subtracted of the product between the area covered by cells (A) and the mean fluorescence of background readings (B), i.e., CTCF = ID – (A × B).

#### Vascular Network Formation (VNF) in dECM Hydrogels

ASC and human pulmonary microvascular endothelial cells (HPMEC) were co-cultured inside the hydrogels to determine their 3D VNF potential. The HPMEC [HPMEC-ST1.6R (Krump-Konvalinkova et al., 2001), kind gift of dr. Unger, Johannes-Gutenberg University, Mainz, Germany] were seeded on gelatin-coated plates (1% gelatin solution in PBS) at a density of 35,000 cells/cm^2^ and cultured until confluency in endothelial culture medium composed of RPMI-1640 basal medium (#BE04-558F, Lonza, Basel, Switzerland) with 10% heat-inactivated fetal bovine serum (FBS; #F0804, Sigma-Aldrich, Missouri, United States), 1% penicillin/streptomycin (#15140122, Gibco Invitrogen, Carlsbad, USA), 1% L-glutamine (#17-605E, Lonza Biowhittaker, Verviers, Belgium), 5 U/mL heparin (LEO Laboratories Limited, Ballerup, Denmark), and 50 g/mL bovine brain extract (BBE, in-house preparation). Cells were kept at 37°C with a minimum relative humidity of 95% and 5% CO_2_.

For the determination of 3D VNF, ASC and HPMEC were seeded inside the three different pre-gels at a final density of 1 × 10^6^ per mL, at an ASC:HMVEC ratio of 1:2. Pre-gels were prepared as described and adjusted to 1x DMEM by addition of 1/10th of the volume of 10x DMEM. After pre-gels were mixed with the cells, 200 μL of the suspension was pipetted inside wells of an 8-well Chamber Slide™ System (ibidi GmbH, Gräfelfing, Germany) and incubated at 37°C for 1 h. Upon gelation, 200 μL endothelial culture medium was added per well. Cells were cultured for 7 days and medium refreshed every 2 days.

After PBS washes, cell-loaded dECM hydrogels were fixed with 4% PFA in PBS for 1 h, washed with PBS twice, frozen in liquid nitrogen, cryosections (50 μm) were deposited on microscope slides. Samples were permeabilized with 0.5% Triton X-100 in PBS for 15 min and blocked with 1% BSA and 5% donkey serum solution in PBS for 15 min. To assess 3D VNF, samples were incubated overnight with rabbit anti-SM22α (1:400; #ab14106, Abcam, Cambridge, UK) for detection of ASC-derived pericytes (Hajmousa et al., 2018; Terlizzi et al., 2018) and mouse anti-human PECAM-1 (1:100, #M0823, Dako, Glostrup, Denmark) to detect endothelial cells, washed with 0.05% Tween in PBS and incubated with donkey anti-rabbit IgG (H+L) Alexa Fluor® 594 (1:400; #A-21207, Life Technologies, Carlsbad, United States) and donkey anti-mouse IgG (H+L) Alexa Fluor® 488 (1:400; #ab150105, Abcam, Cambridge, UK) at RT for 1 h. Nuclei were stained with DAPI (4 μg/ml; #D9542-5MG, Sigma-Aldrich, Missouri, United States). Confocal laser scanning microscope (TCS SP8, Leica Microsystems, Wetzlar, Germany) was used to acquire Z-stack images at 20x magnification. 3D vascular network formation was evaluated qualitatively in 3D reconstructed images post-processed using ImageJ 3D viewer plugin, and quantitatively by the fluorescence intensity of PECAM-1 labeling. In our experience, ASC do not express PECAM-1 when cultured in the endothelial culture medium, thus this marker solely visualized vascular-like structures in our system.

### Statistical Analysis

All data were obtained from at least three independent experiments. Data are presented as the mean ± standard error of the mean (SEM). Graphs and statistical analysis were done using GraphPad Prism (Version 6.01; GraphPad Software, Inc., La Jolla, United States). Differences between and among groups were analyzed by Student's *t*-test, One-way ANOVA with Sidak's multiple comparison test or Two-way ANOVA with Holm-Sidak multiple comparison test.

## Results

### Decellularization of Cardiac Tissue Efficiently Removed Cellular Constituents and Maintained Extracellular Matrix Structure and Components

**Figure 2:**
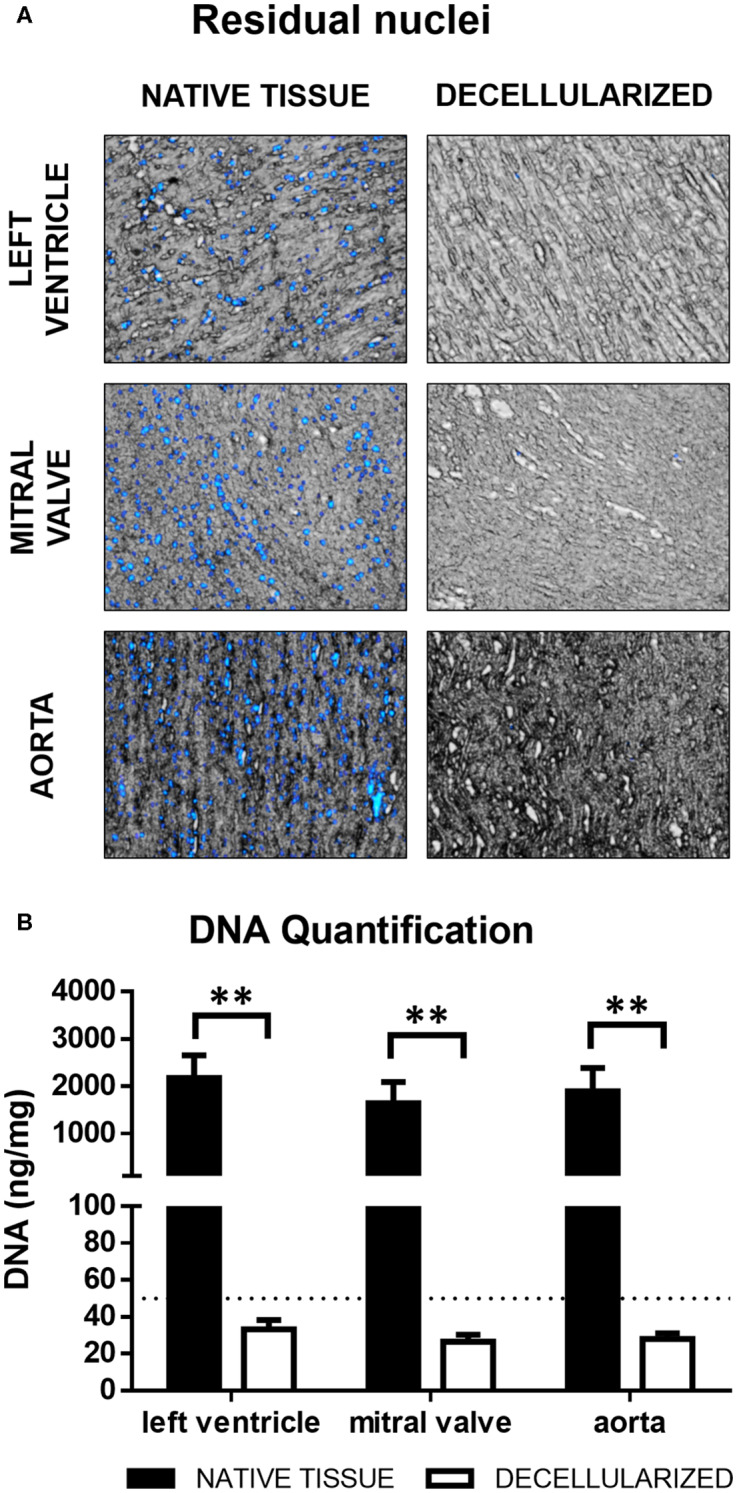
**(A)** Residual nuclei. Fluorescence overlay on brightfield. **(B)** DNA quantification. **p < 0.01. The dotted line represents the maximum accepted DNA concentration for decellularized tissues. Data derived from three independent experiments.

**Figure 3:**
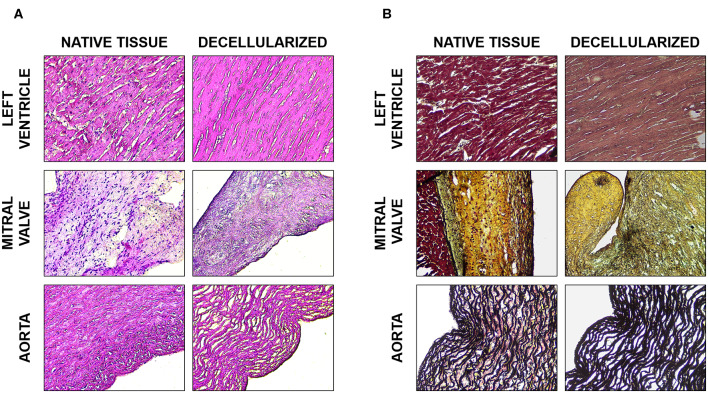
Histological analysis. **(A)** H&E staining of the native and decellularized left ventricle, mitral valve, and aorta tissues. **(B)** Movat's pentachrome stain of the native and decellularized left ventricle, mitral valve, and aorta tissues; muscle in dark red, collagen and reticular fibers in yellow, nuclei and elastin fibers in black, GAGs in blue, and fibrin in bright red.

Porcine cardiac components i.e., left ventricle, mitral valves, and aorta were subjected to a rigorous decellularization procedure to generate cell-free extracellular matrix (dECM). The presence of remaining DNA in the decellularized ECM was assessed both by detection of nuclei with DAPI and by quantification of residual genomic DNA. For all three tissues, only rare disperse nuclei could be detected after decellularization (Figure 2A). The same finding was corroborated in Hematoxylin/Eosin-stained sections (Figure 3A) and Movat's Pentachrome stainings (Figure 3B). Genomic DNA quantification showed a near 99% reduction (Figure 2B, *n* = 3), in all three types of ECM compared to native tissue. The dECMs contained <50 ng/mg of DNA per dry weight, the standard maximal value for decellularized tissues (Crapo et al., 2011).

**Figure 4:**
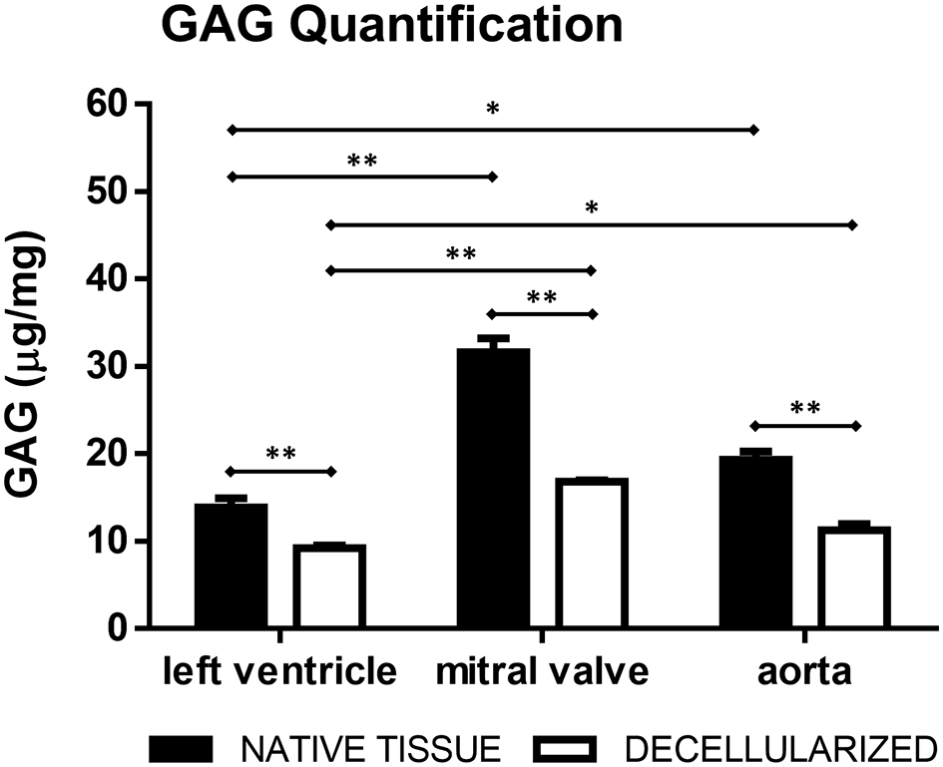
GAG quantification in the native and decellularized left ventricle, mitral valve, and aorta tissues (μg/mg of dry weight). *p < 0.05 and **p < 0.01. Data derived from three independent experiments.

Hematoxylin/Eosin (Figure 3A) and Movat's pentachrome (Figure 3B) stained sections showed that the ECM organization was unaffected i.e., comparable to controls (native tissue, Figure 3). The original tissues, and consequently the dECM, presented diverse GAG content: mitral valves contained little more than 30 μg/mg of GAGs, aorta about 20 μg/mg and left ventricular myocardium about 15 μg/mg. The final percentage of GAGs content in the dECM was more than fifty percent of that present in the original tissue for all the three tissues (Figure 4).

### Decellularized Cardiovascular Tissues Differ in Protein Composition

**Figure 5:**
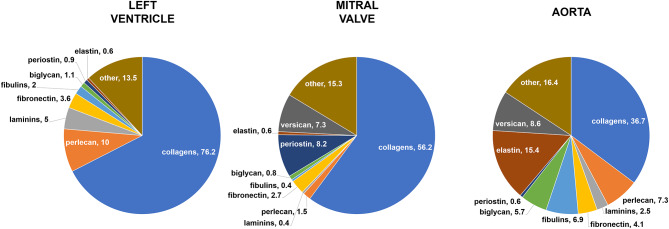
Mass spectrometry analysis. Relative protein abundance (% of ECM spectra). The sum of the parts may be less than or greater than 100% because this graphical representation takes into account only the mean values, without standard deviations. Data derived from three independent experiments.

The protein composition of the extracellular matrix from the three cardiovascular tissues (left ventricle, mitral valve, and aorta) was investigated with mass spectrometry. A total of 64 different ECM proteins was identified and their spectral percentage i.e., the proportional number of molecules of each ECM protein in the samples is given in Table S1. A summary of the results is in Figure 5.

The proteomics analyses (*n* = 3) showed that all three tissues contained collagens as their main ECM component, representing around 76% of left ventricle dECM, 56% of mitral valve dECM, and 37% of aortic dECM. The detailed distribution of the 64 proteins, including collagen types, is described in Table S1. When different types and isoforms of collagens were analyzed separately, it was demonstrated that ventricular ECM presented higher amounts of collagen III alpha-1 chain, collagen IV alpha-1 chain, collagen IV alpha-2 chain, and collagen VI alpha-2 chain when compared to both valvar and aortic ECM. A significant difference was also demonstrated for collagen VI alpha-3 chain, such as it was more expressed in the valvar ECM, corresponding to the main ECM protein in this tissue. Elastin, in turn, was found in expressive quantity in the aortic tissue (~15%), but not in the other two cardiovascular ECM, in which it was found only in scanty amounts (<1%). Another fibrillar ECM protein found in mass spectrometry analysis was fibronectin, representing between 2.7 and 4.1% of all the ECM proteins for all the three tissues, without significant differences among them.

Laminins, a basement membrane constituent, were found in a less expressive, but still representative, amount. The ventricular ECM was the one presenting the higher quantity of laminins (5%), while the valvar ECM was the one presenting the less amount (0.4%), with an intermediate percentage for the aortic dECM (2.5%). The analysis of different isoforms of laminins showed significant differences for laminin subunit alpha-2 and for laminin subunit gamma-1, such as these isoforms were more expressed in the ventricle ECM than in the other cardiovascular tissues. Perlecan, another basement membrane constituent, was also found in a significant higher amount in ventricular (10%) and aortic (7.3%) ECM, when compared to the valvar (1.5%) ECM. Other main proteoglycans found in the three cardiovascular ECM were versican and biglycan. Versican was found in similar amounts in both the valvar (7.3%) and aortic (8.6%) ECM, but interestingly not found in the ventricular ECM. Finally, biglycan (a TGF-β co-receptor) was significantly more expressed in the aortic (5.7%) ECM than in the other two cardiovascular tissues, representing around 1% for the ventricle and mitral valve dECM.

Other ECM proteins found were fibulins, periostin, cartilage intermediate layer protein 2 (CILP-2), elastin microfibril interfacer 1 (EMILIN-1), latent-TGF-β-binding protein, and lysyl oxidase. Fibulins represented an important amount of the proteins found in cardiovascular ECM, particularly in the aortic tissue. The three types of fibulins found in mass spectrometry were fibulin-1, fibulin-2, and fibulin-5, such as the first two were found in lesser amount—and in similar distribution among the tissues—than the last. Fibulin-5, in turn, was expressed in a significantly higher amount in the aortic tissue (6.9%) when compared to both other cardiovascular ECM (<2% for both). Periostin was found in considerable amounts (8.2%) in the valvar ECM, but was almost non-existent in the other two ECM (<1%), although this difference did not reach statistical significance. CILP-2 was found in considerable quantity in the valvar tissue (3.7%), but not in ventricular or aortic ECM. EMILIN-1, a glycoprotein responsible for the interface between elastin and microfibrils, was only found in the aortic ECM, similarly to lysyl oxidase, an extracellular copper-dependent enzyme responsible for the cross-linking between collagen and elastin (and also between collagen fibers), which was found in greater amount in the aortic ECM (1.4%). Finally, latent-TGF-β-binding protein, which regulates TGF-β bioavailability, was also only found in the aortic ECM.

### Hydrogels Derived From the Left Ventricle, Mitral Valve, and Aorta dECM Vary in Structural Morphology and Mechanical Properties

**Figure 6:**
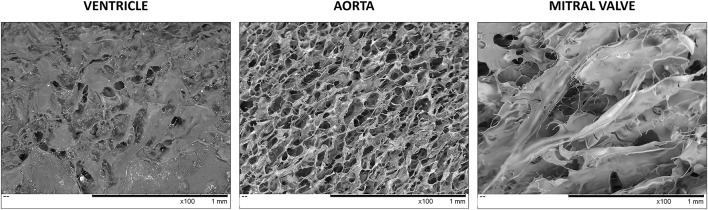
Scanning electron microscopy of the left ventricular, mitral valve, and aortic dECM hydrogels.

Scanning electron microscopy (SEM) demonstrated that the three hydrogels present considerably different structural morphology (Figure 6). The ventricle-derived hydrogel showed a pattern resembling a dense grouping of plate-like structures. Aorta-derived hydrogels, in turn, exhibited a network of honeycomb-like pores. Finally, mitral valve-derived hydrogels displayed another distinct pattern, with *millefeuille*-like sheets of extracellular matrix allocated in several very thin layers.

**Figure 7:**
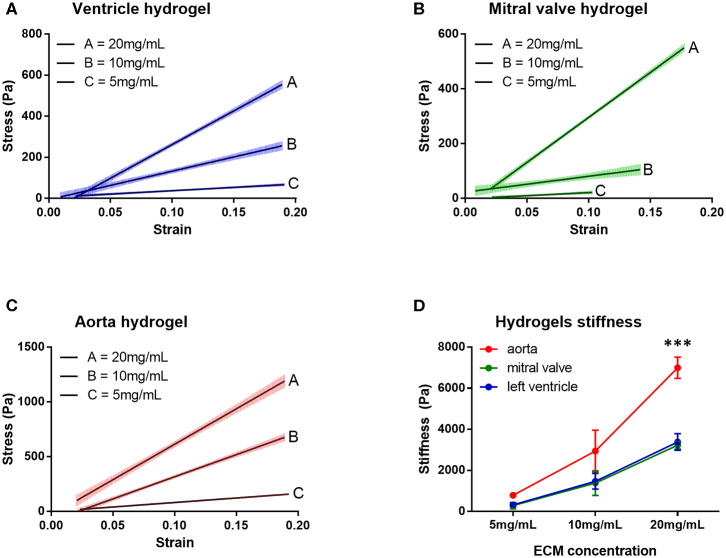
Stress vs. strain curves measured at a strain rate of 0.2 s^−1^ for the three hydrogels at different concentrations: **(A)** ventricle hydrogel, **(B)** mitral valve hydrogel, and **(C)** aorta hydrogel. **(D)** Hydrogels stiffness of the three hydrogels in different concentrations. ***p = 0.0001. Data derived from three independent experiments.

The stress vs. strain curves for the hydrogels derived from the three cardiovascular dECM were plotted for hydrogels with 5, 10, and 20 mg/mL dECM concentration and stiffness was calculated as the slope of the stress vs. strain curve (Figure 7, Table 1, *n* = 3). The stiffness increased, for all tissues, with the increase of ECM concentration (Two-way ANOVA, concentration factor, *p* < 0.0001). For 5 and 10 mg/mL ECM concentrations, no statistically significant differences were found among the hydrogels. For 20 mg/mL ECM concentration, however, hydrogels derived from aortic ECM proved to be significantly stiffer than both the ventricular and valvar hydrogels (Two-way ANOVA, hydrogel factor, *p* = 0.0002; Holm-Sidak multiple comparison test: left ventricle vs. aorta *p* = 0.0001, mitral valve vs. aorta *p* = 0.0001).

**Table 1:** Mechanical properties of the three different hydrogels.

	**Left ventricle**				**Mitral valve**			**Aorta**		
**STIFFNESS**										
Concentration (mg/mL)	5	10	20		5	10	20	5	10	20
Stiffness (Pa)	325 ± 149	1473 ± 663	3384 ± 698		289 ± 222	1386 ± 1039	3233 ± 323	792 ± 75	2942 ± 1755	6998 ± 895
**VISCOELASTICITY**										
Element	E1	E2	E3	E4	E1	E2	E3	E1	E2	E3
T(s)	0.24 ± 0.02	1.96 ± 0.06	13.16 ± 0.49	198.50 ± 31.16	0.23 ± 0.02	2.56 ± 0.33	27.12 ± 11.70	0.26 ± 0.11	2.47 ± 1.13	25.22 ± 18.02
Ei	1169 ± 163	689 ± 140	508 ± 119	822 ± 202	1417 ± 222	567 ± 149	683 ± 142	4814 ± 995	1131 ± 608	671 ± 218
RI (%)	36.9 ± 2.6	21.5 ± 0.4	16.1 ± 1.1	25.5 ± 1.5	60.6 ± 12.1	23.5 ± 1.3	23.9 ± 5.5	74.9 ± 10.4	17.6 ± 8.9	11.2 ± 4.5

*τ, relaxation time constant; E_i_, spring constant; RI, relative importance*.

**Figure 8:**
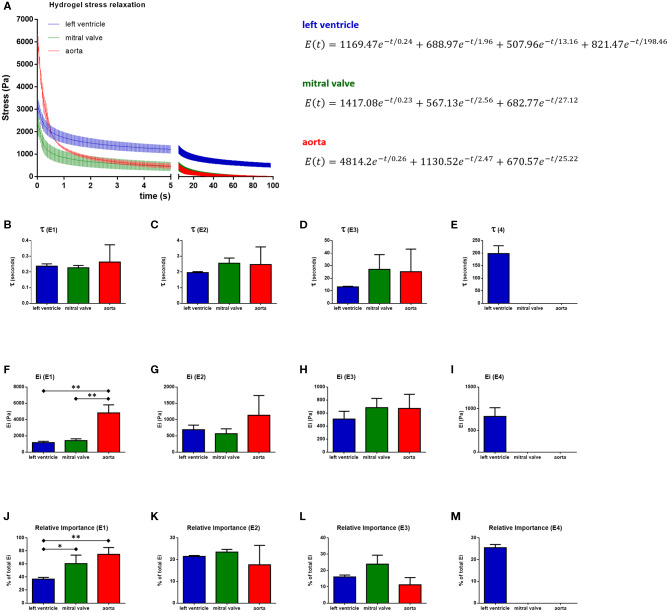
Stress relaxation behavior of 20 mg/ml hydrogels and the average Maxwell model fitting output. **(A)** Hydrogels stress relaxation curves and their respective averaged Maxwell model equations. Relaxation time constants (*t*), spring constants (*E*_*i*_), and relative importances (RI) are shown for the first **(B,F,J)**, second **(C,G,K)**, third **(D,H,L)**, and fourth **(E,I,M)** Maxwell elements. *p < 0.05 and **p < 0.01. Data derived from three independent experiments.

The stress relaxation over time was plotted and the generalized Maxwell model was fitted to the data. Each hydrogel was described according to their relaxation time constants (*t*), spring constants (*E*_*i*_), and relative importance (Figure 8, Table 1, *n* = 3). The stress relaxation curves showed that left ventricular hydrogels started with a low stress value, but kept higher residual stress, while the aortic hydrogels started with a higher stress value and presented an important relaxation, reaching zero within the 100 s of follow-up. Mitral valve hydrogels, in turn, started with low stress values, relaxed quickly reaching zero within the 100 s. This indicates that ventricular hydrogel behaved more like viscoelastic solid, whereas the aorta and valvar hydrogel like a viscoelastic liquid.

Left ventricle hydrogel resolved in four Maxwell elements, while mitral valve and aortic hydrogels resolved in three Maxwell elements to fit to measured stress relaxation. For all the three hydrogels, the first Maxwell element presented the highest impact, representing around 37, 51, and 76% of the relaxation in, respectively, the ventricular, valvar, and aortic hydrogels. The relative importance of the second Maxwell element varied between 18 and 23% among the hydrogels, while these values were between 11 and 24% for the third Maxwell element. The fourth Maxwell element represented a significant relative importance for the ventricular hydrogels, of around 26%. The Maxwell elements, for all hydrogels, were defined according to their *t* values. The first Maxwell element showed different *E*_*i*_ and relative importance (*RI*_*i*_) values among the hydrogels. *E*_*i*_ values were higher for the aortic hydrogel when compared to both the left ventricular and mitral valve hydrogels (One-way ANOVA, *p* = 0.0005; Holm-Sidak multiple comparison test: left ventricle vs. aorta *p* = 0.0009, mitral valve vs. aorta *p* = 0.0009). The first Maxwell element had a stronger impact in aortic hydrogels compared to left ventricle hydrogels, with the valvar hydrogels showing a relative importance value in between (One-way ANOVA, *p* = 0.0087; Holm-Sidak multiple comparison test: left ventricle vs. aorta *p* = 0.0093, left ventricle vs. mitral valve *p* = 0.0492). The *E*_*i*_ or *RI*_*i*_ of the second and the third Maxwell element did not differ.

### Hydrogels Derived From the Left Ventricle, Mitral Valve, and Aorta dECM Differ in Bioactivity

**Figure 9:**
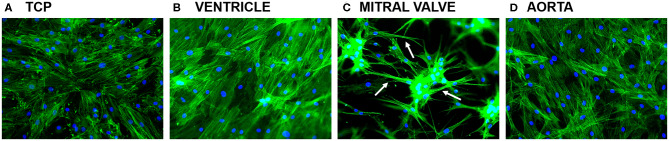
ASC cultured for 7 days on top of tissue culture plastic (TCP), left ventricular, mitral valve, and aortic dECM hydrogels stained for phalloidin. **(A)** TCP, **(B)** ventricle, **(C)** mitral valve, and **(D)** aorta.

After 7 days of culture, ASCs had formed confluent monolayers on tissue culture plastic (TCP), and on the left ventricle and aortic dECM hydrogels (Figures 7A,B,D). In these cases, the ASC had actin filaments throughout their cytoplasm. On mitral valve dECM hydrogel, ASCs had aggregated and formed long cytoplasmic protrusions (Figure 9C, arrows).

**Figure 10:**
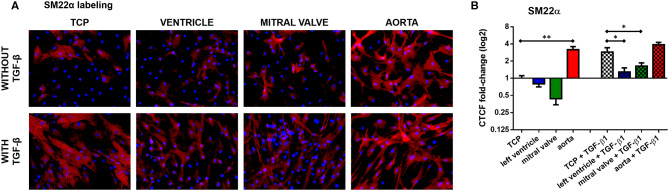
ASC myogenic differentiation on the hydrogels (concentration: 20 mg/mL). **(A)** SM22α staining. **(B)** Quantification of SM22α expression. *p < 0.05 and **p < 0.01. Data derived from three independent experiments.

Our earlier research showed that TGF-β1 stimulation efficiently differentiates ASCs into smooth muscle cells (SMC). Seven days of TGF-β1 stimulation, on TCP indeed differentiated ASC in SM22α-expressing SMC (Figure 10, *n* = 3), while only few ASC normally expressed SM22α. In contrast, TGF-β1 stimulation of ASCs on left ventricular dECM hydrogels differentiated the cells when compared to non-induced ASC, but appeared to be blocked when compared to SMC differentiation in TCP, as evidenced by the reduced expression of SM22α (One-way ANOVA, *p* = 0.0001; Holm-Sidak multiple comparison test: TCP + TGF-β1 vs. left ventricle + TGF-β1 *p* = 0.0108). Similarly, on mitral valve dECM hydrogels, TGF-β1-induced SMC differentiation of ASCs was also blocked and SM22α expression was reduced when compared to cells induced in TCP (One-way ANOVA, *p* = 0.0001; Holm-Sidak multiple comparison test: TCP + TGF-β1 vs. mitral valve + TGF-β1 *p* = 0.0400). In contrast, non-induced ASC differentiated to SMC on aortic dECM hydrogels (One-way ANOVA, *p* < 0.0001; Holm-Sidak multiple comparison test: TCP vs. aorta *p* < 0.0001). The additional stimulation with TGF-β1 had no measurable influence.

### Left Ventricular, Mitral Valve, and Aortic dEMC Hydrogels Differentially Influence VNF

**Figure 11:**
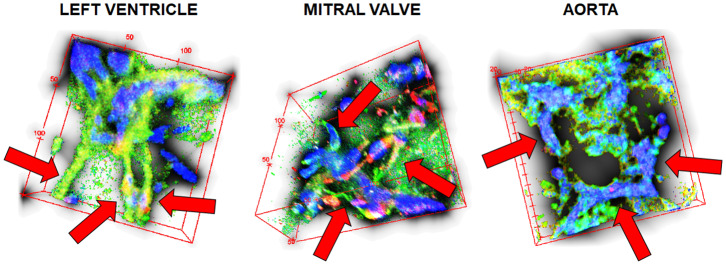
3D vascular networking formation in the three different dECMs hydrogels. Stacked 3D image reconstruction of 50 μm sections. Blue, nuclei; green, HPMEC; red, ASC. Red arrows point to the vessel-like structures within each micrograph.

Vascular network formation was evaluated in 3D by confocal microscopy in the three different hydrogels. After 7 days of co-culturing ASC and HPMEC (1:2) inside dECM hydrogels, VNF had occurred in the left ventricle, mitral valve and aortic hydrogels (Figure 11) albeit that the network morphologies differed. In left ventricle dECM hydrogels, vascular structures appeared to be both thicker and longer (Figure 11, red arrows) compared to both other hydrogels. In mitral valve and aortic dECM hydrogels, vascular structures were fewer and smaller (Figure 11). In all three gels, large aggregates of cells had formed as observed by nuclear staining with DAPI.

## Discussion

The main results of our investigations are that we optimized a procedure to reproducibly and reliably generate extracellular matrix hydrogels from decellularized, highly different cardiac components i.e., left ventricle, mitral valve, and aorta. We show that the left ventricle, mitral valve, and aorta dECM hydrogels differ in largely molecular composition, mechanical properties and bioactivity (Table 2). The major findings were that left ventricle dECM hydrogels (1) contained the highest content of basement membrane constituents; (2) had viscoelasticity comprising four Maxwell elements and around 3 kPa stiffness (20 mg/mL gel), (3) suppressed ASC differentiation, but (4) augmented VNF. Mitral valve dECM hydrogels, on the other hand, had (1) periostin as a major constituent; (2) a lower viscoelastic complexity (three elements) and also around 3 kPa stiffness (20 mg/mL gel); (3) suppressed ASC differentiation, and (4) had marginal VNF support. Finally, aortic dECM hydrogels differed from both others and (1) had the highest content of non-collagen constituents i.e., elastin, fibulins, and biglycan to mention a few; (2) a viscoelasticity of three elements with high stiffness, around 7 kPa (20 mg/mL gel); (3) caused non-induced ASC differentiation to SMC; and (4) marginally supported VNF.

**Table 2:** Molecular and biomechanical differences among ECM from different tissues and the respective biological response.

	**Molecular differences**	**Biomechanical differences**	**Biological response**
Left ventricle	↓ GAG ↑Collagen III alpha-1 chain ↑Collagen IV alpha-1 chain ↑Collagen IV alpha-2 chain ↑Collagen VI alpha-2 chain ↑Laminin subunit alpha-2 ↑Laminin subunit gamma-1 ↓Versican	↑Maxwell elements	1. Confluent monolayer cell growth 2. Inhibition of TGF-β1-induced myogenic differentiation 3. Substantial vascular network formation
Mitral valve	↑GAG ↑CILP-2 ↑Collagen VI alpha-3 chain ↓Perlecan		1. Clustered cell growth 2. Inhibition of TGF-β1-induced myogenic differentiation 3. Limited vascular network formation
Aorta	↑Biglycan ↑EMILIN-1 ↑Fibulin-5 ↑Latent TGF-β-binding protein 2 ↑Lysyl oxidase	↑Stiffness	1. Confluent monolayer cell growth 2. Non-induced myogenic differentiation 3. Limited vascular network formation

*↑Greater expression/value than the other two dECM*.

*↓Lower expression/value than the other two dECM*.

These biochemical and biomechanical differences among the three different cardiovascular tissue-derived hydrogels correlates to the differences found in the physiological situation, i.e., the biochemical and biomechanical properties of the respective tissues. These differences, as shown in the present work, directly impact cell behavior and, thus, are of great relevance because they can be used to mimic the *in vivo* microenvironment during *in vitro* studies. In fact, the use of such tissue-specific microenvironments for tissue engineering has been proposed to reduce the use of animal models during drug development and pathophysiological studies, besides tissue-engineering for therapeutic purposes (Liguori et al., 2017).

### Molecular Clues

In regard to the molecular clues, the first to be noted is the differences in GAG content. GAG are responsible for the binding of several growth factors that can stimulate cell response—such as fibroblast growth factors (FGF), hepatocyte growth factor (HGF)—chemokines as interleukins, enzymes (and their inhibitors), as well as several ECM proteins including fibronectin, laminin, and type V collagen (Varki et al., 1999; Esko et al., 2017). Although in ventricular tissue the reduced GAG content followed the reduced content of the versican core protein, in valvar tissue GAG expression was increased compared to the other matrices besides the reduced content of the perlecan core protein. This shows that not only GAG content may vary among the matrices, but also the types of proteoglycans which are present in different tissues, which might influence how these GAGs interact with the cells. Previously, it was shown that extrinsic perlecan suppressed adipogenic and promoted osteogenic differentiation in bone marrow mesenchymal stem cells (Nakamura et al., 2014). Although the influence of perlecan on myogenic differentiation was never investigated, it is known that this proteoglycan binds the latent form of TGF-β, increasing the growth factor bioavailability (Chen et al., 2007; Sengle et al., 2011). Versican, in turn, facilitates chondrocyte differentiation *in vivo* by binding to TGF-β and fixating it to the ECM, thus, favoring TGF-β signaling pathways in the surrounding cells (Choocheep et al., 2010). *In vitro*, versican was shown to induce myogenic differentiation of fibroblasts by increasing active TGF-β signaling (Carthy et al., 2015).

The second difference in the molecular composition of the ECM refers to the increased presence of collagens type III, IV, and VI in the ventricular tissue when compared to the valvar and arterial matrices. The absence of type III collagen was shown to be responsible for the myofibroblast differentiation in a mice model, suggesting that this type of collagen plays an important role in maintaining the fibroblastic phenotype (Volk et al., 2011). Interestingly, collagen VI—which was increased in both the ventricular and valvar matrices—was shown to be responsible for cardiac fibroblast differentiation into myofibroblasts *in vitro* (Naugle et al., 2006). Paradoxically, collagen VI was also shown to enhance MSC expansion while maintaining the stem cell phenotype (Smeriglio et al., 2017). One of the reasons why collagen VI seems to be involved in cell fate might be related to the biophysical modulation of the environment—either in the native ECM or in a hydrogel—since it was shown that collagen VI is crucial for the biomechanical integrity of the pericellular matrix in MSC (Twomey et al., 2014) and also that soluble fragments of collagen VI can mediate stimulation of DNA synthesis via tyrosine phosphorylation of paxillin, FAK, and p130CAS—proteins that associate with focal adhesions—in the absence of classical growth factors (Rühl et al., 1999).

Laminin family was another group of proteins which was differentially expressed among the matrices. Ventricular ECM presented increased levels of laminin subunit alpha-2 and laminin subunit gamma-1 when compared to the other two ECM types. Laminins have been shown to suppress chondrogenic, but to induce osteogenic differentiation of mesenchymal stem cells, contributing to bone tissue development, through an ERK-dependent pathway (Klees et al., 2005, 2008; Hashimoto et al., 2006; Mittag et al., 2012). Other effects of laminins on mesenchymal stem cells related to ERK pathways are the differentiation into insulin-producing cells (Lin et al., 2010), and the promotion of neurite outgrowth (Mruthyunjaya et al., 2010). In regard to myogenic differentiation, laminin-2 has been suggested to induces smooth muscle myogenesis by down-regulation of RhoA, but no other studies investigated the effect of laminins on myogenic differentiation (Beqaj et al., 2002). Herein, we did not find the expected myogenic differentiation supposedly promoted by laminins, in fact, the opposite was demonstrated. This might be related to the fact that several other factors were acting on the ASC and the strength of laminin influence was not enough to exercise any effect. On the other hand, laminin was shown to promote 3D vascular network formation in collagen scaffolds by regulating VEGF uptake (Stamati et al., 2014), what corroborates our findings. We demonstrated that ventricular dECM-derived hydrogel, which present higher laminin content than the other two matrices, was also the one showing better 3D vascular network formation.

Mitral valve extracellular matrix showed increased protein expression of cartilage intermediate layer protein 2 (CILP-2), which was absent in both other tissues, although cartilage intermediate layer protein 1 (CILP-1) was present in both the ventricular and valvar matrices. This is an interesting finding that coincides with the behavior of the adipose stromal cells in regard to differentiation upon TGF-β1 stimulation. Cartilage intermediate layer protein is a large secreted glycoprotein and its CILP-1 form has been shown to block TGF-β1 activity through direct interaction with TGF-β1 and inhibition of TGF-β1 signaling (Seki et al., 2005; van Nieuwenhoven et al., 2017; Zhang et al., 2018). Although there are no studies in regard to the CILP-2 interaction with TGF-β1, it may probably present a similar behavior to CILP-1.

The aortic dECM hydrogels, in turn, showed a series of proteins significantly higher expressed compared to the two other matrices, all of them being proteins constituents of elastic fibers. One of them was biglycan, a proteoglycan that forms complexes with collagen and elastic fibers components and binds to TGF-β1 (Hildebrand et al., 1994; Reinboth et al., 2002). This, *per se*, might justify the contribution of the aortic matrix to the non-induced myogenic differentiation of ASC. In fact, the use of the word non-induced was preferred over spontaneous because it is known FBS-containing culture medium contains 1–2 ng/mL of TGF-β1 (Danielpour et al., 1989; Oida and Weiner, 2010). Thus, what might actually be in course is the binding of serum-derived TGF-β1 to the hydrogel and, consequently, its presentation to the cells in a higher concentration than that found in the culture medium. Another protein highly expressed in aortic ECM was elastin microfibril interfacer 1 (EMILIN-1), a protein responsible for the formation of the elastic fiber and for cell anchoring. EMILIN-1 has been shown to inhibit TGF-β signaling *in vivo* (Zanetti et al., 2004; Zacchigna et al., 2006), what could be understood as paradoxical to our findings. However, it was demonstrated that the mechanism of TGF-β signaling inhibition by EMILIN-1 occurs through the binding with the precursors of TGF-β (proTGF-β), not allowing it to be converted into mature TGF-β (Zacchigna et al., 2006). A third protein which was more abundantly present in the aortic matrix was fibulin-5. Fibulin-5 is an ECM protein crucial for elastogenesis and also able to promote elastic fiber organization (Hirai et al., 2007). The role of fibulin-5 is closely related to the fact this protein contains several Arg-Gly-Asp (RGD) motifs (Albig and Schiemann, 2005), besides also presenting calcium-binding EGF-like domains (Timpl et al., 2003). While the RGD motifs bind the integrins of cells, the calcium-binding EGF-like domains of fibulin-5 were shown to bind latent TGF-β-binding protein 2 (LTBP-2) (Hirai et al., 2007), another protein we found in significantly higher levels in the aortic matrix. The LTBP-2, in turn, plays an important role in TGF-β availability in the cell microenvironment by the binding latent form of TGF-β which is, then, cleaved by proteases into mature TGF-β (Robertson et al., 2015). Since ASC produce TGF-β (Rehman et al., 2004; Du et al., 2016), released in the latent form, LTBP-2 may increase TGF-β availability by binding the latent TGF-β in the ECM, not allowing it to be diluted in the medium and, then, also increasing the concentration of the growth factor available to the cells. Finally, the last protein found to be overexpressed in the aortic matrix, when compared to the ventricular and mitral matrices, was lysyl oxidase (LOX), an enzyme that initiates the crosslinking in collagen and elastin. LOX is known to both bind TGF-β (Atsawasuwan et al., 2008), but also to have its expression upregulated by the growth factor (Gacheru et al., 1997; Shanley et al., 1997).

### Biomechanical Clues

Besides the different molecular clues given by the three different matrices, we also investigated the influence of their biomechanical properties, which differs for each matrix and may play a role in cell behavior. In summary, the two main findings in this regard were a significantly higher stiffness found in the hydrogels derived from aortic dECM and viscoelastic solid nature of hydrogels derived from ventricular dECM, which is also manifested as a higher number of Maxwell elements. Stiffness is known to influence cell behavior, including cell differentiation and microvascular network formation, being extensively discussed in literature (Engler et al., 2006; Even-Ram et al., 2006; Wells, 2008; Califano and Reinhart-King, 2009; Park et al., 2011; Schaap-Oziemlak et al., 2014; Ye et al., 2015; Mao et al., 2016). Viscoelasticity, on the other hand, is a combination of influencing factors much less debated. In general, authors treat their hydrogel materials as elastic when, in fact, they are viscoelastic due to large water content. This means that, besides elasticity, which is generally represented by stiffness, these materials present another important biomechanical property which is viscoelasticity, represented by stress relaxation.

Hydrogels with specific stiffnesses were previously shown to promote mesenchymal stem cells differentiation into smooth muscle cells under TGF-β induction, although non-induced differentiation was not described (Park et al., 2011). In fact, stiffnesses ranging between 8 and 17 kPa are considered to promote myogenic differentiation (Engler et al., 2006), which is very close to our findings for the hydrogels derived from aortic dECM (6.9 ± 0.8 kPa). This might be a contributing factor for the behavior of ASC cultured on aortic dECM hydrogels, particularly when taken together with the previously discussed biomolecular clues found in this matrix. Hydrogels constituted of pure collagen exhibit lower stiffness values in comparison to dECM hydrogels, ranging from 30 Pa for 3 mg/mL to 1.8 kPa for 20 mg/mL (Cross et al., 2010). This difference might be related to the combination of collagen fibers with other ECM proteins, such as elastin, present in dECM hydrogels, which importantly improve their elastic modulus. In regard to vascular network formation, literature is limited but it has been shown that soft substrates facilitate endothelial cell network assembly (Califano and Reinhart-King, 2008, 2009), which corroborates with our findings.

In regard to the stress relaxation, to the best of our knowledge there is no more than a handful of studies in literature describing the effects of stress relaxation on cell behavior, particularly regarding cell spreading (Cameron et al., 2011; McKinnon et al., 2013; Chaudhuri et al., 2015, 2016; Bauer et al., 2017; Charrier et al., 2018), proliferation (Cameron et al., 2011; Chaudhuri et al., 2016; Bauer et al., 2017), and differentiation (Cameron et al., 2011, 2014; Chaudhuri et al., 2016; Charrier et al., 2018). Unfortunately, the order of magnitude in stress relaxation varies among the studies, from *t*_1/2_ *(*time until 50% relaxation) starting at 60 s (Chaudhuri et al., 2016) until *t*_1/2_ of more than 30,000 min (Cameron et al., 2011). Chaudhuri et al. demonstrated that while osteogenic differentiation requires fast-relaxing hydrogels (optimal when *t*_1/2_ = 60 s), adipogenic differentiation requires slow-relaxing hydrogels (optimal when 50% relaxation *t*_1/2_ = 2,300 s) (Chaudhuri et al., 2016). Probably, myogenic differentiation would require a relaxation time between these two. Although Cameron *et al*. showed improved myogenic differentiation in hydrogels presenting *t*_1/2_ = 250 min (15,000 s) when compared to hydrogels with even higher *t*_1/2_ (up to over 30,000 min) (Cameron et al., 2011), they also showed that both adipogenic and osteogenic differentiation were increased in these hydrogels with lower *t*_1/2_ values, what lead to questioning if the range of stress relaxation time used by the authors was not much above the ideal for cell culturing. The stress relaxation in our hydrogels, in contrast, was even faster than those reported by Chaudhuri et al., for all the three different matrices, with *t*_1/2_ lower than 1 s.

Another difference between the studies was the stiffness of these hydrogels. Although both authors kept the stiffness constant within their experiments, Chaudhuri et al. used hydrogels with 9 and 17 kPa (Chaudhuri et al., 2016), while Cameron et al. used hydrogels of 4.7 kPa (Cameron et al., 2011). Thus, there is also the combinatory effect of stiffness and stress relaxation, which is the whole point of the viscoelasticity theory. In our study, for instance, stiffness was not controlled, so that hydrogels could present high values of stiffness while presenting low stress relaxation time (aorta), low values of stiffness with also low stress relaxation time (mitral valve), or low values of stiffness with high stress relaxation times (left ventricle). This points to the need for a more robust descriptive reference for viscoelasticity than only looking at stiffness and stress relaxation separately, which we believe to be the Maxwell elements. As previously mentioned, these elements allow the dissection of several main components exerting the resistive forces during and after strain.

In the present study, we showed that the hydrogels derived from ventricular dECM, which led to the inhibition of ASC differentiation but supported the growth of robust and interconnected vascular networks in 3D, presented an extra Maxwell element. To define what component is being represented by each Maxwell element is a difficult task in natural materials (Peterson et al., 2013), particularly because it is not possible to control the components present in these materials. It is, however, well accepted that the first element, which tends to present a *t-*value smaller than 1s, represents water and soluble small molecules (Bausch et al., 1999; Cense et al., 2006; Peterson et al., 2013). The second element, with a *t-*value between 1s and 10s, could represent glycosaminoglycans, small proteoglycans, and other small ECM proteins (e.g., biglycan, EMILIN-1, fibulin-5) and water bound to GAGs and other hygroscopic molecules, while the third, with a *t-*value between 10s and 100s, could be large extracellular matrix proteins (e.g., fibrillar collagens, versican, perlecan, laminin). Finally, the fourth Maxwell element, which was only present in the hydrogels derived from ventricular dECM, should then be related to some component either only present in the ventricular ECM or at least increased in this matrix when compared to the other two. Thus, considering the increased expression and the percentual protein content in the ventricular ECM (see Table S1), the fourth Maxwell element should be collagen IV.

Differently from fibrillar collagens (e.g., collagens type I, II, and III), collagen IV is network-forming collagen (Muiznieks and Keeley, 2013). It was previously shown that while crosslinked meshes of synthetic polymers increase the time required for stress relaxation, linear chains lead to faster stress relaxation (Chaudhuri et al., 2016; Charrier et al., 2018). This behavior found in synthetic polymers could also explain the findings of our study so that the crosslinked meshes of collagen IV are leading to the prolonged time necessary for stress relaxation. To date, no other study investigated the effect of viscoelasticity on cell differentiation or vascular network formation under the lens of Maxwell elements, so that the present work is the first to do so. Previous authors, however, hypothesized on how the viscosity i.e., stress relaxation may influence cell behavior. In our opinion, the next step is relating cell behavior to the strength and relaxation time constants of individual Maxwell elements, which are in turn related to the hydrogel composition. Briefly, while in elastic matrices the forces exerted by the cell on the matrix (and consequently by the matrix on the cell) are kept constant over time, in viscoelastic materials the matrix relaxes over time so that the forces are decreased and the matrix is remodeled (Chaudhuri et al., 2016). Faster relaxation i.e., faster matrix remodeling would lead to increased RGD-ligand clustering, cell shape change, proliferation, and differentiation stimuli (Chaudhuri et al., 2016).

## Limitations

A limitation of the study is the use of CTCF, i.e., fluorescence intensity, to quantify the myogenic markers. Ideally, the quantification of the number of cells expressing the marker would be a preferable alternative. However, the fact that, as demonstrated in Figure 9, the mitral valve hydrogel tended to lead cells to form clusters, precludes running a cell to cell analysis.

## Conclusion

The three main cardiovascular tissues (myocardium, valves, and vessels) can be used to fabricate hydrogels from dECM. Different tissues, however, can lead to different cell behavior, in regard to differentiation and vascular network formation, by combining different molecular and biomechanical clues. Our results motivate further studies on how hydrogels derived from different matrices (beyond cardiovascular tissue) can guide the behavior of different cell types.

## Data Availability Statement

The datasets generated for this study are available on request to the corresponding author.

## Author Contributions

GL and TL: conceptualization, data curation, formal analysis, investigation, methodology, writing—original draft, and writing—review and editing. SM, VS, VT, and JD: investigation. PS and LM: supervision, validation, and writing—review and editing. MH: conceptualization, funding acquisition, project administration, resources, supervision, validation, and writing-review and editing.

## Conflict of Interest

The authors declare that the research was conducted in the absence of any commercial or financial relationships that could be construed as a potential conflict of interest.
