# Supplementary material for: Molecular and Biomechanical Clues From Cardiac Tissue Decellularized Extracellular Matrix Drive Stromal Cell Plasticity
Source: Front Bioeng Biotechnol. 2020 May 29;8:520. doi: 10.3389/fbioe.2020.00520 (PMC7273975; doi:10.3389/fbioe.2020.00520)
Supplement: Supplementary file 1 [file Table_1.DOCX]

**SUPPLEMENTARY MATERIAL**

| **Table S1. Extracellular matrix protein content (% of ECM spectra)** | | | | |
| --- | --- | --- | --- | --- |
| **protein** | **left ventricle** | **mitral valve** | **aorta** | **p-value (One-Way ANOVA)** |
|  | **mean±SD** | **mean±SD** | **mean±SD** |  |
| **aggrecan core protein** | 0.0±0.0 | 0.6±0.5 | 0.0±0.0 | 0.0795 |
| **asporin** | 0.5±0.6 | 0.3±0.5 | 0.3±0.2 | 0.8514 |
| **biglycan** | 1.1±0.9 | 0.8±0.8 | 5.7±3.1 | 0.0352 |
| **cartilage intermediate layer protein 1** | 0.9±0.8 | 0.6±0.6 | 0.0±0.0 | 0.2542 |
| **cartilage intermediate layer protein 2** | 0.0±0.0 | 3.7±1.3 | 0.0±0.0 | 0.0012 |
| **collagen I alpha-1 chain** | 8.7±9.7 | 1.5±0.9 | 5.7±3.4 | 0.3939 |
| **collagen I alpha-2 chain** | 9.5±8.7 | 11.6±10.7 | 8.0±3.1 | 0.8674 |
| **collagen II alpha-1 chain** | 0.1±0.2 | 0.0±0.0 | 0.0±0.0 | 0.7461 |
| **collagen III alpha-1 chain** | 6.9±0.4 | 0.3±0.3 | 1.1±0.6 | <0.0001 |
| **collagen IV alpha-1 chain** | 8.4±3.7 | 0.0±0.0 | 0.5±0.1 | 0.0055 |
| **collagen IV alpha-2 chain** | 3.3±0.4 | 0.1±0.1 | 0.6±0.3 | <0.0001 |
| **collagen IV alpha-6 chain** | 0.1±0.2 | 0.0±0.0 | 0.0±0.0 | 0.5111 |
| **collagen V alpha-1 chain** | 0.5±0.2 | 0.4±0.2 | 0.3±0.3 | 0.4893 |
| **collagen V alpha-2 chain** | 1.3±0.9 | 0.7±0.2 | 0.2±0.2 | 0.1103 |
| **collagen V alpha-3 chain** | 0.1±0.2 | 0.0±0.0 | 0.0±0.0 | 0.4219 |
| **collagen VI alpha-1 chain** | 1.9±1.1 | 3.0±2.2 | 2.4±1.1 | 0.6802 |
| **collagen VI alpha-2 chain** | 15.8±1.5 | 6.7±0.8 | 6.1±3.1 | 0.0019 |
| **collagen VI alpha-3 chain** | 17.7±6.1 | 30.8±5.4 | 10.7±4.4 | 0.0096 |
| **collagen VI alpha-5 chain** | 0.0±0.0 | 0.4±0.4 | 0.0±0.1 | 0.1348 |
| **collagen VIII alpha-1 chain** | 0.6±0. | 0.1±0.0 | 0.0±0.0 | 0.1203 |
| **collagen XI alpha-1 chain** | 0.1±0.2 | 0.0±0.0 | 0.0±0.0 | 0.4219 |
| **collagen XII alpha-1 chain** | 0.0±0.0 | 0.1±0.1 | 0.0±0.0 | 0.1279 |
| **collagen XIV alpha-1 chain** | 0.7±0.8 | 0.2±0.2 | 0.3±0.3 | 0.5118 |
| **collagen XV alpha-1 chain** | 0.1±0.2 | 0.1±0.1 | 0.4±0.4 | 0.3205 |
| **collagen XVI alpha-1 chain** | 0.0±0.0 | 0.2±0.2 | 0.0±0.0 | 0.1288 |
| **collagen XVIII alpha-1 chain** | 0.0±0.0 | 0.0±0.0 | 0.1±0.1 | 0.4219 |
| **collagen XXI alpha-1 chain** | 0.3±0.3 | 0.0±0.0 | 0.0±0.0 | 0.1286 |
| **collagen XXVIII alpha-1 chain** | 0.2±0.3 | 0.0±0.0 | 0.0±0.0 | 0.4219 |
| **decorin** | 0.3±0.2 | 0.6±0.8 | 0.5±0.5 | 0.7372 |
| **dermatopontin** | 0.0±0.0 | 0.3±0.3 | 0.3±0.1 | 0.1960 |
| **EGF-containing fibulin-like ECM protein 1** | 0.7±0.6 | 0.0±0.0 | 0.8±0.2 | 0.0791 |
| **elastin** | 0.6±0.6 | 0.6±0.6 | 15.4±23.7 | 0.3728 |
| **elastin microfibril interfacer 1 (EMILIN-1)** | 0.0±0.0 | 0.0±0.0 | 0.3±0.2 | 0.0101 |
| **fermitin family homolog 2** | 0.2±0.2 | 0.0±0.0 | 0.5±0.6 | 0.2449 |
| **fibrillin-1** | 0.5±0.3 | 0.2±0.2 | 1.4±1.4 | 0.2725 |
| **fibrillin-2** | 0.0±0.0 | 0.0±0.0 | 0.6±1.1 | 0.4219 |
| **fibromodulin** | 0.1±0.2 | 0.7±0.6 | 0.9±0.2 | 0.0793 |
| **fibronectin** | 3.6±1.0 | 2.7±2.4 | 4.1±2.9 | 0.7645 |
| **fibulin-1** | 0.1±0.2 | 0.0±0.1 | 0.0±0.0 | 0.5625 |
| **fibulin-2** | 0.2±0.2 | 0.0±0.0 | 0.0±0.0 | 0.0975 |
| **fibulin-5** | 1.7±1.1 | 0.4±0.1 | 6.9±1.8 | 0.0014 |
| **hyaluronan and proteoglycan link protein 1** | 0.0±0.1 | 0.1±0.1 | 0.3±0.2 | 0.1290 |
| **hyaluronan and proteoglycan link protein 3** | 0.0±0.0 | 0.0±0.0 | 0.0±0.1 | 0.4219 |
| **laminin subunit alpha-2** | 0.3±0.1 | 0.0±0.0 | 0.0±0.0 | 0.0004 |
| **laminin subunit alpha-3** | 0.0±0.0 | 0.0±0.0 | 0.0±0.1 | 0.4219 |
| **laminin subunit alpha-4** | 0.1±0.2 | 0.0±0.0 | 0.6±0.6 | 0.1374 |
| **laminin subunit beta-1** | 0.7±0.4 | 0.1±0.1 | 0.3±0.1 | 0.0711 |
| **laminin subunit beta-2** | 1.8±1.3 | 0.1±0.1 | 0.6±0.2 | 0.0968 |
| **laminin subunit gamma-1** | 2.1±0.7 | 0.2±0.2 | 0.9±0.3 | 0.0052 |
| **latent-TGF-β-binding protein 1** | 0.0±0.0 | 0.0±0.0 | 0.0±0.1 | 0.4219 |
| **latent-TGF-β-binding protein 2** | 0.0±0.0 | 0.0±0.0 | 0.8±0.5 | 0.0250 |
| **lumican** | 3.6±0.9 | 3.8±5.7 | 1.3±0.6 | 0.6208 |
| **lysyl oxidase (protein-lysine 6-oxidase)** | 0.0±0.0 | 0.1±0.1 | 1.4±0.7 | 0.0100 |
| **lysyl oxidase-like (lysyl oxidase homolog 1)** | 0.0±0.0 | 0.0±0.0 | 1.0±1.2 | 0.2073 |
| **nidogen 1** | 0.3±0.3 | 0.0±0.0 | 0.0±0.0 | 0.1160 |
| **nidogen-2** | 0.3±0.3 | 0.1±0.1 | 0.9±0.5 | 0.0598 |
| **periostin** | 0.9±0.8 | 8.2±7.5 | 0.6±0.5 | 0.1268 |
| **perlecan** | 10.0±0.6 | 1.5±1.0 | 7.3±3.5 | 0.0075 |
| **prolargin** | 0.4±0.4 | 0.1±0.1 | 0.9±0.4 | 0.0665 |
| **tenascin** | 0.2±0.2 | 0.2±0.2 | 0.0±0.0 | 0.2520 |
| **TGF-β-induced protein ig-h3** | 1.8±1.2 | 1.1±1.1 | 0.2±0.1 | 0.1780 |
| **thrombospondin 1** | 0.0±0.0 | 0.2±0.2 | 0.0±0.0 | 0.0803 |
| **thrombospondin 4** | 0.2±0.3 | 0.0±0.0 | 0.0±0.0 | 0.2151 |
| **versican core protein** | 0.0±0.0 | 7.3±2.4 | 8.6±4.1 | 0.0181 |
